# Immunophenotypic Profile of Adult Glioblastoma IDH-Wildtype Microenvironment: A Cohort Study

**DOI:** 10.3390/cancers16223859

**Published:** 2024-11-18

**Authors:** Sofia Asioli, Lidia Gatto, Uri Vardy, Claudio Agostinelli, Vincenzo Di Nunno, Simona Righi, Alicia Tosoni, Francesca Ambrosi, Stefania Bartolini, Caterina Giannini, Enrico Franceschi

**Affiliations:** 1 Department of Biomedical and Neuromotor Sciences (DIBINEM), University of Bologna, 40127 Bologna, Italy; sofia.asioli3@unibo.it (S.A.); caterina.giannini@mayo.edu (C.G.); 2 IRCCS Istituto delle Scienze Neurologiche di Bologna, 40139 Bologna, Italy; 3 Nervous System Medical Oncology Department, IRCCS Istituto delle Scienze Neurologiche di Bologna, 40139 Bologna, Italy; vincenzo.dinunno@ausl.bologna.it (V.D.N.); a.tosoni@isnb.it (A.T.); stefania.bartolini@isnb.it (S.B.); enricofra@yahoo.it (E.F.); 4 School of Medicine and Surgery, University of Bologna, 40138 Bologna, Italy; uri.vardy@studio.unibo.it; 5 Haematopathology Unit, IRCCS Azienda Ospedaliero-Universitaria of Bologna, Via Massarenti 9, 40138 Bologna, Italy; claudio.agostinelli@unibo.it; 6 Department of Medical and Surgical Sciences, University of Bologna, 40138 Bologna, Italy; 7 Pathology Unit, Maggiore Hospital-AUSL Bologna, 40133 Bologna, Italy; simona.righi5@unibo.it (S.R.); francesca.ambrosi3@unibo.it (F.A.); 8 Department of Medical and Surgical Sciences (DIMEC), University of Bologna, 40126 Bologna, Italy; 9 Department of Laboratory Medicine and Pathology, Mayo Clinic, Rochester, MN 55905, USA

**Keywords:** glioblastoma, tumor microenvironment, TME, tumor-associated macrophages, tumor-infiltrating lymphocytes, TILs

## Abstract

**Background**: Glioblastoma *IDH*-wildtype (GBM *IDH*-wt) is the most aggressive brain tumor in adults and is characterized by an immunosuppressive microenvironment. Different factors shaping its tumor microenvironment (TME) regulate tumor progression and treatment response. The aim of this study was to characterize the main immunosuppressive elements of the GBM *IDH*-wt TME. **Methods:** Immunohistochemistry for CD3, CD4, CD8, CD163, programmed death ligand 1 (PD-L1) and programmed death 1 (PD1) was performed on surgical tumor specimens from patients diagnosed with GBM *IDH*-wt, according to the CNS WHO 2021 criteria. The impact of categorical variables on time-dependent outcomes such as overall survival (OS) and progression-free survival (PFS) has been estimated through the Kaplan–Meier method. **Results:** We included 30 patients (19 males and 11 females), median age of 59.8 years (range 40.2–69.1 years). All patients underwent surgery followed by temozolomide concurrent with and adjuvant to radiotherapy. MGMT was methylated in 14 patients (47%) and unmethylated in 16 patients (53%). The overall absolute percentages of CD4+ lymphocytes, both intratumoral and perivascular, were significantly more represented than CD8+ lymphocytes in the TME (*p* = 0.02). A low density of CD4+ lymphocytes (≤10%) was found to be a favorable prognostic factor for GBM outcome (*p* = 0.02). Patients with MGMT methylated and unmethylated tumors exhibited a distinct TME composition, with a significant higher number of perivascular CD8+ lymphocytes (*p* = 0.002), intratumoral CD8+ lymphocytes (*p* = 0.0024) and perivascular CD4+ lymphocytes (*p* = 0.014) in MGMT unmethylated tumors. PD-L1 expression in tumor cell surface was observed in four tumors (13.3%), and PD1 expression in infiltrating T lymphocytes was observed in nine (30%) tumors, with predominantly perivascular distribution. **Conclusions:** MGMT methylated and unmethylated tumors exhibit different immune profiles, likely reflecting the different biology of these tumors. The expression of PD-L1 in GBM *IDH*-wt patients is confined to a small subpopulation. While we found a significant association between low CD4+ lymphocyte density (≤10%) and survival, given the small numbers of our cohort, the prognostic value of CD4+ lymphocyte density will need to be validated in large-scale studies.

## Simple Summary

Glioblastoma *IDH*-wildtype (GBM *IDH*-wt) is the most common primary brain tumor in adults, characterized by a severe immunosuppressive milieu, with very limited therapeutic options. The efficacy of immunotherapy in GBM is still under investigation; thus, it is critically important to investigate the immunomodulatory mechanisms acting within the GBM microenvironment. We aimed to perform an immunohistochemical characterization of a panel of immune biomarkers (CD3, CD4, CD8, CD163, programmed death ligand 1 and programmed death 1) of 30 GBM patients to determine the tumor immune infiltrate and the distribution of the principal immunological markers.

## 1. Introduction

GBM *IDH*-wt is the most common and aggressive primary brain tumor in adults. Despite the adoption of multimodal treatments, GBM *IDH*-wt represents one of the greatest challenges in neuro-oncology with a median survival of less than two years and a recurrence rate exceeding 90% [1,2].

T-cell-based immunotherapy has prolonged the survival of patients with solid cancers, but despite significant progress in the past decade, it has proven to be an effective strategy only in small subsets of cancer patients [3].

GBM *IDH*-wt is a tumor with an immunosuppressive microenvironment [4], and, for a long time, it has been considered an “immunological desert”, poorly infiltrated by T lymphocytes, and with a unique immune-escape ability. Through a broad range of mechanisms resulting in the T-cell loss of effector function, senescence, anergy and exhaustion, GBM *IDH*-wt often induces a state of global T-cell dysfunction and active immunosuppression, sustained by the expression of several inhibitory receptors [5,6,7]. Nonetheless, some observations have emerged from clinical immunotherapy studies, highlighting that even GBM *IDH*-wt is permissive for a continuous T-cell infiltration and activation and could be responsive to the action of immunotherapy [8,9,10,11].

The GBM *IDH*-wt TME exhibits a high level of intratumoral heterogeneity and is composed of a complex system of cells that display diverse functions in response to different stimuli, where lymphocyte and monocyte lineage cells are characterized by dynamic changes, having both effector and suppressive phenotypes and, at alternate moments, pro-and anti-tumor properties [12,13,14,15]. In this context, tumor-infiltrating lymphocytes (TILs) and tumor-associated macrophages (TAMs) are considered dominant cellular subpopulations [12,16]. TILs participating in anti-tumor immunity in GBM *IDH*-wt include CD4^+^ and CD8^+^ [17,18] and are key players in the host immune response to tumor [19]. While they have shown a favorable impact on survival in patients with breast, colorectal and ovarian cancer, their prognostic role in GBM *IDH*-wt patients is still indeterminate [20,21,22,23].

Tumor-associated macrophages (TAMs) are crucial infiltrating immune cells that interact with glial cells, accounting for 30–40% of the cellular components in GBM *IDH*-wt [24,25], and include microglia, perivascular macrophages, meningeal macrophages and macrophages of the circumventricular organ and choroid plexus. TAMs exhibit substantial diversity and plasticity with a “double-edged” function depending on their polarization: they are classified into two main groups, respectively named “classically activated macrophages” (CD68+ M1 cells) and “alternatively activated macrophages” (CD163+ M2 cells). M1 macrophages, characterized by CD68 expression, produce free radicals that can lead to DNA damage with the potential to contribute to tumoricidal activity [26]. In contrast, M2 macrophages, characterized by both CD68 and CD163 expression, are considered to promote tumor growth and metastasis by releasing chemokines, which are inflammatory growth factors [26]. In particular, M2 macrophages are characterized by the high expression of the scavenger receptor CD163, which plays a role in the clearance of haptoglobin/hemoglobin complexes [27]. CD163+ TAMs exhibiting an M2 phenotype are associated with poor prognosis in breast cancer, melanoma and other solid tumors [28,29,30]. M2-polarized TAMs are highly represented in the GBM *IDH*-wt TME exerting an anti-inflammatory action and contributing to the cold immunosuppressive state of this tumor [31,32].

The variety of immune cells, the expression of immunomodulatory targets and the synergetic mechanisms of TAMs and TILs still remain controversial and poorly investigated in GBM [33].

The aim of this study was to characterize the main immunosuppressive elements of the GBM IDH-wt TME.

### The Unique Immune Inhibitory Phenotype of GBM

Immunotherapy, ranging from check point inhibitors to oncolytic viruses, tumor-derived vaccines and chimeric-antigen receptor T cells, is promising for many solid tumors including GBM [33,34,35,36]. However, advancements in treating GBM are scarce and slow. This is due to several factors: first the lack of well-characterized targetable neoantigens; second, the “cold” TME that hampers the generation of sustained and productive immunologic responses [37]. The elements that make GBM as a “cold” unique immune environment are multiple, including the contribution of immune cells to suppress both the innate and adaptive immunity, the immune inhibitory proteins expressed by GBM and the immunosuppression induced by chemotherapy and radiotherapy.

On the side of the innate immune system, the basic nonspecific line of defense in non-immunized subjects, the first aspect to consider is the blood–brain barrier, a physical barrier characterized by intercellular tight junctions, which controls the permeability of the endothelium and selectively restricts the passage of immune cells and molecules into the brain [8,38]. Other essential factors that regulate innate immunity in GBM include pattern recognition receptors (PRRs), eosinophils, Natural Killer (NK) lymphocytes, macrophages and dendritic cells [39]. PRRs are proteins expressed on the cell membrane of dendritic cells, macrophages, monocytes, neutrophils and endothelial cells that recognize pathogens through two classes of molecules: Pathogen-Associated Molecular Patterns (PAMPs), expressed by pathogenic microbes, and Damage-Associated Molecular Patterns (DAMPs), expressed by host cells during cell damage or cell death [39,40]. PRR activation triggers the formation of the inflammasome, a multiprotein complex enabling a potent and fast secretion of inflammatory cytokines, resulting in cell death. Receptors belonging to the Toll-like family (TLR) and Nucleotide-binding Oligomerization Domain (NOD)-like receptors (NLRs) constitute the paradigm of PRRs. TLRs are transmembrane receptors mainly expressed on the membrane of macrophages and dendritic cells that recognize specific structures typical of pathogens and microbial agents; this recognition activates the immune responses of the sentinel cells, inducing the production of inflammatory cytokines, such as interleukin-1 (IL-1) and tumor necrosis factor (TNF). These molecules, at a systemic level, through IL-6, act on the liver, inducing the production of acute phase proteins [39]. The dysregulation of TLR and NLR signaling is very common in GBM, and the over-regulation of PRRs is correlated with tumor progression, angiogenesis and poor prognosis [40]. For example, the overexpression of TLR-4 in GBM patients is associated with unfavorable outcome [41,42], and many reports [43] have confirmed that GBM progression is associated with the upregulation of NOD-like receptors expressed by macrophages.

Eosinophils are innate immune cells, regulators of allergy and eosinophilia, that exhibit close relations with the process of tumorigenesis and that are proved to be associated with the prognosis of some solid tumors [44]. In particular, they are associated with a favorable prognosis in many cancers [45] including GBM as well as nasopharyngeal cancer [46] and oral squamous cell carcinoma [47]. Interestingly, glioma incidence is inversely associated with allergic comorbidities such as asthma [48], characterized by a high level of eosinophils. Thus, an eosinophil-enriched immune infiltrate appears to be a protective factor against the development and progression of gliomas. Recent research has shown that glioma incidence is significantly suppressed in the presence of elevated levels of circulating eosinophils [49], and a “tumor tissue eosinophilia” often represents a favorable prognostic factor [50]. Most likely, eosinophils through their cytotoxic function, via Fas ligand, block GBM proliferation and induce apoptosis [47].

NK cells and cytotoxic CD8^+^ are the first barrier against microorganisms and tumor cells [51]; these two very different types of cells have a powerful cytotoxic activity orchestrated by an intricate network of inhibitory and activating signals that converge in the release of cytotoxic proteolytic granules, leading to apoptosis. While the NK cells are a population of innate lymphoid cells, the CD8^+^ cell is one of the major components of adaptive immunity [51]. Cancer cells are detected via neoantigen presentation on their major histocompatibility complex (MHC) class I molecules, resulting in targeted removal by circulating NK or CD8+ T cells [52]. Increasing evidence has suggested that GBM tumor cells sometimes downregulate the expression of MHC class I molecules, critical for “self” versus “non-self” distinction, thereby escaping detection by the host’s innate immune system [33]. The loss of MHC class II, critical for antigen presentation to adaptive immune cells, has also been documented in GBM, underscoring the immunosuppressive nature of this tumor [53]. Furthermore, in GBM, the NK and CD8+ response and the adaptive immune system are largely suppressed by the recruitment of abundant immune modulators that make the TME “cold” and refractory to immunotherapy such as Treg, TAM, and immunosuppressive molecules, including IL-6, IL-10 and TGF-β, and enzymes such as indolamine 2,3-dioxygenase (IDO) [51,54]. IDO has an immunosuppressive effect by decreasing the proliferation, function and survival of T cells [55].

CD3 is the defining marker for T cells; therefore, all T cells express CD3. CD3+/CD4+ T cells are commonly divided into Tregs and conventional T helper (Th) cells. Th cells control adaptive immunity against pathogens and cancer by activating other effector immune cells. Treg cells are defined as a population of CD4 + lymphocytes that play a fundamental role in controlling reactivity against self-antigens, inhibiting chronic inflammatory responses and maintaining immune tolerance [56]. Patients with GBM present a dysregulation in the CD4+ T-cell fraction, characterized by an expansion of Tregs and a reduction in CD4+ Th cells. Suppressive activities attributed to Tregs include the inhibition of cytotoxic T cells, the maintaining of self-tolerance, the suppression of allergy, feto-maternal and oral tolerance [56]. No specific markers for Treg lymphocytes have been identified because all the presently used markers (CD25, CTLA-4, GITR, LAG-3 and CD127) are nonspecific; nevertheless, the transcription factor forkhead box P3 (FoxP3) seems to be essential for the development and function of these cells [57,58].

In addition, in GBM, CD4+ T and CD8+ T cells are frequently exhausted and dysfunctional and, therefore, ineffective at tumor control. The phenomenon of “exhaustion” is the consequence of a chronic stimulation of T cells by tumor cells that lead to the upregulation of immune checkpoint markers such as PD-1, LAG-3 and TIGIT on T cells [59], rendering TILs inadequate at exerting an effective anti-tumor immune response.

Together, resident microglia and macrophages in GBM are generally referred to as TAMs [60] that approximately represent 40–50% of the tumor mass. After originating from peripheral blood monocytes, macrophages can differentiate into pro-inflammatory CD68+ M1 phenotypes, which have tumoricidal action, promote Th responses and secrete proinflammatory cytokines (TNF-alpha), or CD163+ M2 phenotypes, associated with immunosuppression and tissue repair, facilitating tumor progression [61]. In GBM, TAMs typically exhibit an M2 phenotype, are recruited to the tumor site by molecules produced by neoplastic and stromal cells and produce mediators that contribute to proliferation, angiogenesis, migration, tissue invasion and dissemination [62]. Obviously, the M1/M2 classification is an oversimplification, and macrophages can display overlapped phenotypes depending on their tissue location and type of signaling [27]. Chemoattractant molecules for TAMs are chemokines such as CCL2, cytokines such as VEGF, PDGF (platelet-derived growth factor) and GM-CSF (granulocyte-macrophage colony-stimulating factor), as well as proteins derived from the degradation process of the extracellular matrix such as fibrinogen and fibronectin.

TAMs are able to produce anti-inflammatory cytokines such as IL-6, IL-10 and TGF-β [63]. IL-6 and IL-10 promote a pro-tumor microenvironment via the JAK2/STAT3 pathway [64,65]. TGF-β enhances immunosuppression through a range of mechanisms including NK-cell and T-cell inhibition, IL-2 downregulation and Treg promotion [66]. In addition, TAMs overexpress ligands for immune checkpoints and upregulate IDO and arginase-1 (ARG-1), responsible for the depletion of essential nutrients for lymphocytes.

Moreover, GBM microenvironment is enriched with GM-CSF, which acts on glioma-infiltrating myeloid cells and promotes immunosuppression through the upregulation of IL-4, an anti-inflammatory cytokine [67].

GBM cells, typically, also exert the direct suppression of adaptive immunity through the minimal expression of neoantigens and the overexpression of numerous immune checkpoint molecules such as PD-L1 or cytotoxic T-lymphocyte-associated protein 4 (CTLA-4) [37].

That might mean that, in GB, the immune response against tumor cells could be more easily supported by innate immunity rather than by adaptive immune responses, antibody or cell-mediated. There is still a lot of work to be carried out to identify high-quality neoantigen targets for generating personalized immunotherapies and to understand mechanisms of resistance and immune escape [68]. Another aspect that should not be underestimated is the possibility that conventional therapies for GBM, radiotherapy and chemotherapy can contribute to the immunosuppressive phenotype of this neoplasm, a finding with important implications for the combination of immunotherapy with standard treatment [69]. The current standard of care for GBM is maximal surgical resection followed by temozolomide concomitant and adjuvant to radiotherapy [59,70]. Patients are also treated with steroids to control brain edema.

In animal models, treated glioma cells resulted in more immunosuppressive than untreated samples as a consequence of the impressive overexpression of immunosuppressive cytokines including IL-4, IL-10, IL-6 and GM-CSF [69]. Temozolomide and dexamethasone are known to influence the immune system, inducing lymphopenia and B and T-cell dysfunction, as well as the upregulation of CTLA-4 [71].

Radiotherapy, which varies from whole brain treatment to stereotactic therapy, induces the secretion of immunosuppressive cytokines such as IL-6 and IL-10 [72]. Recently, a randomized phase II trial comparing two types of radiotherapy for GBM (wide irradiation field versus small irradiation field) reported that OS and PFS were significantly better in the small irradiation group. This is probably due to the fact that a wide radiation may reduce the immune function of a large number of lymphocytes.

In conclusion, combined TMZ, radiotherapy and dexamethasone therapy in GBM patients may induce a persistent lymphocytopenia, which is associated with poorer survival [72].

## 2. Materials and Methods

### 2.1. Study Design

This is a retrospective study aimed at assessing the immune profile of adult patients (≥18 years old) with newly diagnosed GBM *IDH*-wt. We retrospectively identified formalin-fixed paraffin-embedded (FFPE) tumor tissue specimens from 30 adult newly diagnosed GBM *IDH*-wt patients who underwent surgery at our institution.

Inclusion criteria were as follows:

(1) Age ≥ 18 years old;
(2) Diagnosis of GBM *IDH*-wt;
(3) Histological slides/formalin-fixed, paraffin-embedded tissue tumor (FFPE) blocks from the archive available to perform immunohistochemical analysis.

Exclusion criteria were as follows:

(1) Histological diagnosis different from GBMs;
(2) Unavailability of histological slides/formalin-fixed, paraffin-embedded tissue tumor (FFPE) blocks.

All cases were independently re-evaluated by an expert neuropathologist (S.A.). Diagnostic criteria were based on the 2021 WHO Classification of CNS tumors [73]. Only patients with a histological diagnosis of GBM *IDH*-wt were included. Demographical, clinical data, medical records and radiological imaging were reviewed for all patients included in the study. All of the cases were evaluated with an immunohistochemical panel consisting of glial fibrillary acidic protein (GFAP), Olig2, *IDH1R132H*, BRAFV600E, P53 and Ki-67 antibodies. Deletion of CDKN2a/2b was detected by FISH analysis. In addition, an NGS panel for IDH1, TERT, PTEN, BRAFV600E and TP53 was performed.

The primary objective of the study was to quantitatively assess the expression of TILS and TAMs and their regional distribution in GBM samples and to determine the expression of the immune modulator targets PD1 and PDL1. GBM-infiltrating immune cells were identified and typed into myeloid or T cells by immunohistochemistry for CD3, CD4, CD8, CD68 and CD163. We evaluated the expression of the immune modulator targets PD1 and PDL1.

The secondary purpose of the study was to explore the prognostic value of the immune cells and immune targets in newly diagnosed GBM *IDH*-wt patients.

The impact of categorical variables on time-dependent events such as overall survival (OS) and progression-free survival (PFS) has been estimated through the Kaplan–Meier method.

The study was approved by the Ethics Committee of the Azienda Sanitaria Locale di Bologna (protocol number CE09113, Bologna, Italy). This study was conducted in agreement with either the most updated Declaration of Helsinki and all the international and local laws applied to clinical trials and patient protection. The study was conducted according to the principles of the ICH Harmonized Tripartite Guideline for Good Clinical Practice.

### 2.2. Immunohistochemistry

Immunohistochemistry for PD-L1 (clone 22C3, DAKO, Santa Clara, CA, USA), PD1 (clone NAT105, CELL MARQUE, Rocklin, CA, USA), CD3 (clone 2GV6 ventana), CD4 (clone SP35 Ventana), CD8 (clone SP57 Ventana), CD68 (monoclonal mouse anti-human CD68, clone KP1, Agilent, Santa Clara, CA, USA) and CD163 (clone MRQ-26, mouse monoclonal antibody, Novocastra, Wetzlar, Germany) was performed. All immunohistochemical analyses were performed on 2-micron formalin-fixed, paraffine-embedded tissue sections. The most significant paraffin blocks representing the tumor and the perivascular area were selected for each sample. Standard procedure with Ventana automated immunostaining was applied (Ventana XT autostainer; Ventana Medical System, Tucson, AZ, USA). Sections were scored by 3 pathologists (S.A, C.A. and F.A.). TILs were evaluated on the entire slide, and lymphocyte density was evaluated separately at higher magnification (400×) counting positive cells (lymphocytes and macrophages) in 2 mm^2^.

### 2.3. Assessment of Tumor-Infiltrating Lymphocytes

Density of CD4+ and CD8+ subpopulations was evaluated manually, counting the cells in serial sections for all predefined regions and normalized to 2 mm^2^ of tissue [74]. T-cell infiltrate was classified as “low” if ≤10 T cells/mm^2^, “high” if >10 cells/mm^2^ or “negative” if no T cells were present.

The prevalent distribution of TILs was classified as (i) intratumoral; (ii) perivascular; and (iii) both.

### 2.4. Assessment of PD-L1 Expression

**Figure 1:**
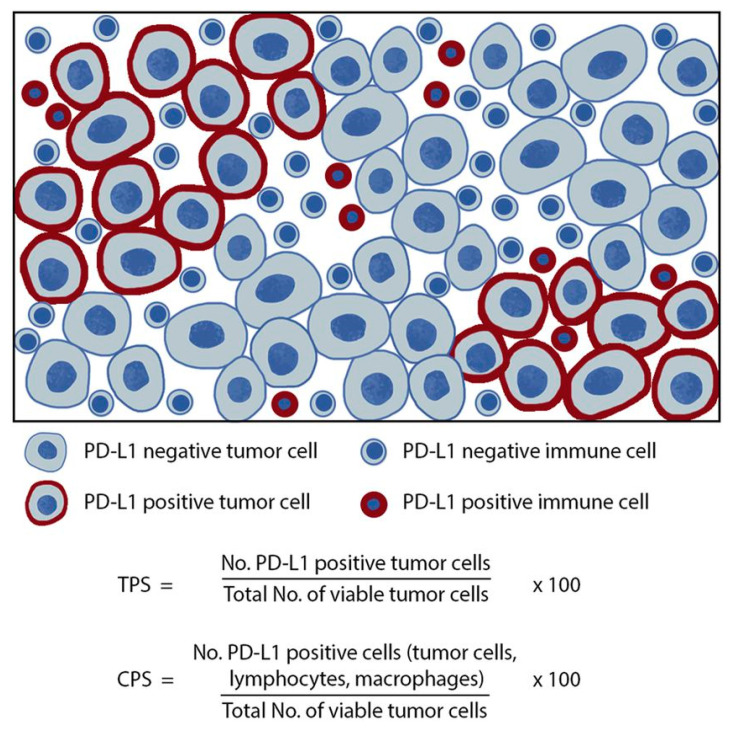
TPS and CPS scoring algorithms for PD-L1. The TPS algorithm is defined as the ratio of PD-L1-positive tumor cells to the total number of viable tumor cells multiplied by 100. The CPS algorithm and is defined as the ratio of PD-L1-positive cells to the total number of viable TC multiplied by 100.

PD-L1 score was evaluated using a “Combined Positive Score” (CPS), consisting of the number of cells with PD-L1 membrane staining (tumor cells, lymphocytes, and macrophages) divided by the total number of viable tumor cells multiplied by 100 [75] (Figure 1). Combined Positive Score (CPS) <1% was considered negative, between 1 and 50% low and >50% high (PD-L1 testing (22C3)—Professional Expert Course Head and Neck Cancer) [76,77].

In addition, Tumor Proportion Score (TPS) assessment was evaluated, too. Tumor Proportion Score (TPS) resulted in the percentage of viable tumor cells showing partial or complete membrane staining at any intensity (Figure 1). TPS was negative if <1%, low within 1% and 50%, or high if >50% [78].

### 2.5. Assessment of PD1 Expression

PD1 expression on T lymphocytes was measured as the number of T lymphocytes that showed immunoreactivity for PD1, and the prevalent distribution was classified as (i) intratumoral, (ii) perivascular or (iii) both.

### 2.6. MGMT Methylation Status Analysis

Analysis of the methylation status of the MGMT gene promoter was performed routinely using the MS-qLNAPCR126 (rapid methylation-sensitive, quantitative PCR assay using Locked Nucleic Acid).

### 2.7. Statistical Analysis

For descriptive purposes, the continuous variables in the study were summarized using the mean, median, standard deviation and range. The categorical variables were summarized using absolute frequency and percentage. The impact of categorical variables on time-dependent events such as overall survival (OS) and progression-free survival (PFS) has been estimated through the Kaplan–Meier method.

Comparisons between curves were made using the log-rank test. To analyze OS as a function of methylation status, type of surgical treatment, chemotherapy and radiotherapy, Cox univariate and multiple regression models were used. For all analyses, the level of statistical significance was *p* < 0.05.

## 3. Results

### 3.1. Patient Cohort

Thirty patients were included, 19 (63.3%) were male, and 11 (36.7%) were female. The median age at diagnosis was 59.8 years (range 40–69 years). Twenty patients underwent subtotal tumor resection (66.7%), nine cases gross total resection (30%) and only one patient a stereotactic biopsy (3.3%). The clinicopathological characteristics of the patients are summarized in Table 1.

All cases showed positivity for GFAP and OLIG2, with expression ranging from focal to diffuse. Immunohistochemical staining with anti-*IDH1R132H* antibody was negative in all cases. The nuclear expression of ATRX was preserved in 21 cases (70%). The overexpression of p53 was present in 9 (of 30) cases (30%). BRAFV600E immunostain was detected in 2 cases (6.6%). The Ki67 proliferation index varied between 15% and 60%. No homozygous deletion of CDKN2a/2b was detected by FISH analysis. By NGS data analysis, *IDH1* and were wild type in all patients (n = 30); 80% of patients harbored TERT mutations (n = 24), 40% exhibited TP53 mutations (n = 12), 6.6% presented BRAF mutations (n = 2) and 6.6% PTEN mutations (n = 2). In particular, the following mutations were found for TERT: C288T in 10 (of 30) cases (33.3%), C124T in 8 cases (26.6%), C146T in 4 cases (13.3%) and C250T in 3 cases (10%). PTEN mutations were identified as p.Ala148Thr in one case and pGly127Ala in another. In two cases (6.6% of patients), the mutation V600E was identified for BRAF. Fourteen cases (47%) exhibited MGMT gene promoter methylation, while 16 cases (53%) were MGMT unmethylated.

After surgery, all patients underwent temozolomide concurrent with and adjuvant to radiotherapy.

The median OS was 13.3 months (95% CI 11.4–20), and the median follow-up was 14.25 months (95% CI 8.98–25.94). Median OS for MGMT methylated patients was 14.9 months; median OS for MGMT unmethylated tumors was 13.3 months (*p*-value = 0.9). Median OS for p53 mutated patients was 15.3 months, and median OS for p53 wt patients was 13.3 months.

### 3.2. Density and Distribution of TILs

TIL populations were characterized according to the expression of CD3, CD4 and CD8. CD3 is the defining marker for T cells: therefore, all T cells express CD3.

In total, 25 (of 30) cases were CD3 positive, 5 (of 30) cases were CD3 negative.

The CD3+/CD4+ lymphocyte count was low (≤10 cells/mm^2^) in 9 cases (30%) and high (>10 cells/mm^2^) in 16 cases (53.3%), compared to 5 negative cases (16.7%). Of the 25 positive cases (83.3%), 10 showed exclusively a perivascular distribution (33.3%) and 14 both a perivascular and intratumoral distribution (46.7%). One case (3.3%) had an exclusively intratumoral distribution.

The CD3+/CD8+ lymphocyte count was low (≤ 10 cells/mm^2^) in 13 cases (43.3%) and high (>10 cells/mm^2^) in 5 cases (16.7%) versus 12 negative cases (40%). Eleven cases (36.6%) showed both perivascular/intratumoral distribution, while seven cases (23.3%) demonstrated an exclusively perivascular distribution.

Overall, T CD4+ cells (intratumoral and perivascular) were numerically more represented than T CD8+ lymphocytes in TME (*p* = 0.02).

### 3.3. Density and Distribution of TAMs

We performed immunohistochemical staining for the monocyte/macrophage markers CD68 and CD163.

CD163, analyzed both intratumorally and perivascularly, was positive in all 30 patients examined. Intratumorally, very high expression was found in 8 (of 30) cases (26.7%), high expression in 7 (23.3%), medium expression in 7 (23.3%) and low expression in 8 (26.7%). CD163 expression at the perivascular level was very high in 8 (of 30) cases (26.7%), high in 8 (26.7%), medium in 7 (23.3%) and low in 7 (23.3%).

**Figure 2:**
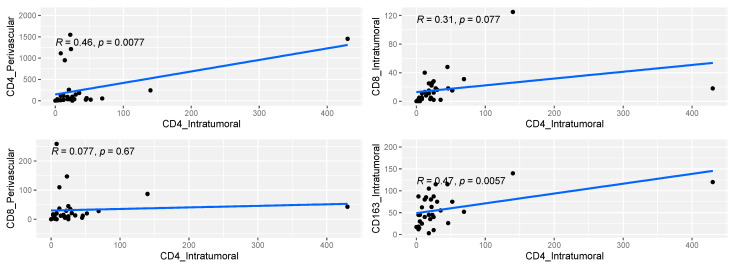

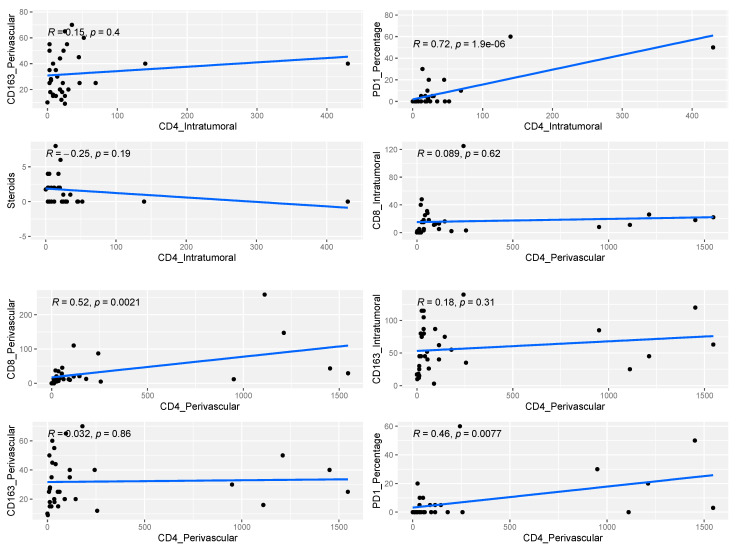
Graphic representation of the linear correlations with 95% confidence interval (grey), *p*−values (*p*) and Pearson/Spearman coefficients (R) between different lymphocyte subpopulations.

Intratumoral CD163+ macrophage density increased in direct proportion to that of intratumoral CD8+ (*p* < 0.001) and intratumoral CD4+ (*p* = 0.006) lymphocytes (Table 2, Figure 2). We observed a positive linear correlation between intratumoral CD163+ lymphocytes density, intratumoral CD8+ (*p* < 0.001) and intratumoral CD4+ (*p* = 0.006) lymphocytes (Table 2, Figure 2).

### 3.4. Expression and Distribution of Tumor-Associated Immunomodulatory Targets

**Figure 3:**
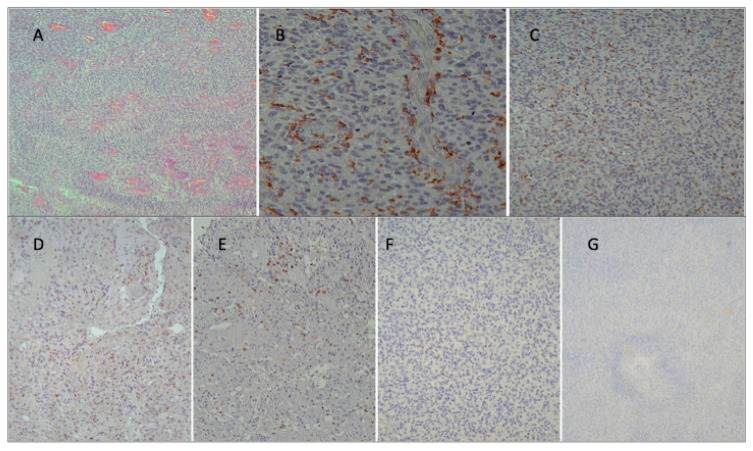
IHC images of a case of our series, showing localization of immune cells in perivascular area and tumor core. (**A**) Hematoxylin and eosin staining; (**B**) CD68+ M1 perivascular; (**C**) CD68+ M1 intratumoral; (**D**) CD4+ perivascular; (**E**) CD8+ perivascular; (**F**) PD-L1 negative; (**G**) PD1 negative.

**Figure 4:**
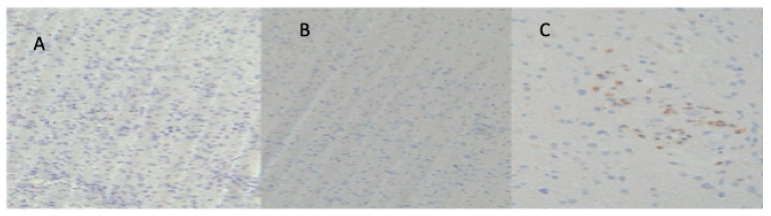
IHC images of a case of our series, showing (**A**) PD1 intratumoral; (**B**) PD-L1 perivascular; (**C**) PD1 perivascular.

PD1+ T cells were present in only nine cases (30%). Among the nine positive cases, seven cases (23.3%) had CPS < 50% and two cases (6.6%) had CPS > 50%. Four cases presented an exclusively perivascular distribution, two perivascular and intratumoral and three exclusively intratumoral (Figure 3 and Figure 4).

PD-L1 was positive in only four cases (13.3%). Two cases (6.6%) had CPS < 50% and two (6.6%) CPS > 50%. Among the four positive cases, two presented a perivascular distribution and two intratumoral distribution (Figure 3 and Figure 4).

### 3.5. TME Heterogeneity Among MGMT Methylated and Unmethylated Tumors

MGMT unmethylated tumors had a significantly higher number of CD8 lymphocytes (both intratumoral and perivascular) and perivascular CD4 lymphocytes in their TME than did methylated tumors (Table 3).

There was no significant difference in the microenvironment composition when tumors were stratified according to the TERT, TP53, BRAF and PTEN mutational statuses.

### 3.6. Survival Analysis

In our cohort median OS for MGMT-methylated patients was 14.9 months; median OS for MGMT unmethylated tumors was 13.3 months (*p*-value = 0.9). Median OS for p53-mutated patients was 15.3 months, and, for p53 wt patients, it was 13.3 months.

Univariate analysis was performed for the following variables, including age (as continuous variable), sex (male vs. female), surgery (complete resection vs. partial resection vs. biopsy), MGMT status (methylated vs. unmethylated), TILS (positive vs. negative), CD4+ density (negative, ≤10%, or >10%), CD8+ density (negative, ≤10%, or >10%) and PDL1 (positive vs. negative).

**Figure 5:**
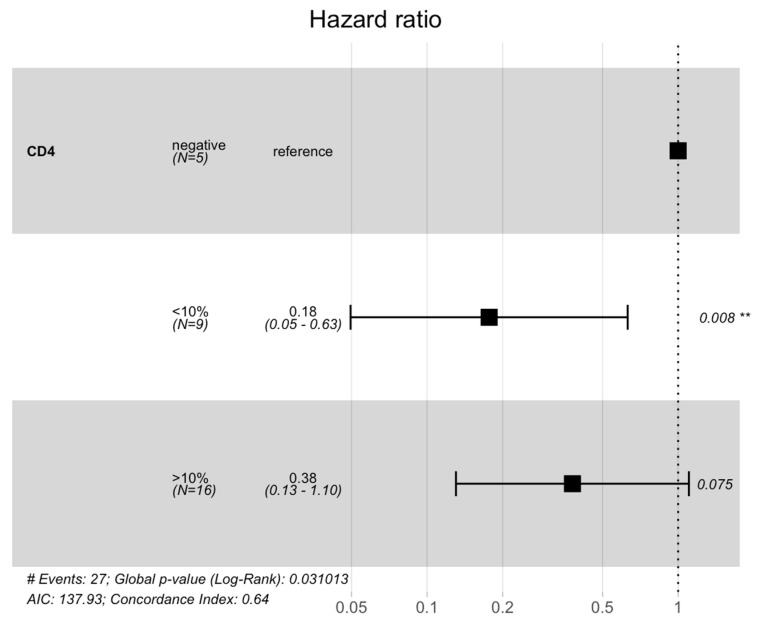
Overall survival according to CD4 percentage. This model incorporates covariates that might influence survival: CD4 infiltrate ≤ 10%, CD4 infiltrate > 10% and CD4 infiltrate negative. Hazard Ratios are visualized with Forest Plots. Patients with low CD4 infiltrate (≤10%) show a statistically significant longer survival (HR = 0.008; *p*-value = 0.02). # stands for “number of events”; ** stands for “statistically significative”.

CD4+ was the only immune variable associated with GBM prognosis in our cohort (Figure 5). In particular, a low CD4+ lymphocyte count (≤10%) was found to be a favorable prognostic factor for GBM outcome (*p* = 0.02) (Figure 5).

The number of CD8+ and CD163+ cells did not correlate with OS or PFS. CD4/CD8 and CD3/CD8 ratio were not related to OS or PFS. No significantly different OS was registered according to PD1 and PDL-1 expression.

## 4. Discussion

TILs, along with resident and infiltrating myeloid cells, make up a significant proportion of the GBM *IDH*-wt TME. The heterogeneity of the cellular composition of GBM *IDH*-wt TME and the complexity of their interactions are a major challenge in the development of successful immunotherapeutic strategies and in the ability to predict their efficacy [79,80]. TME is not only pivotal in tumor progression, but it also contributes to drug resistance; nevertheless, the variety of lymphoid cell types within GBM still remains poorly investigated.

The aim of the study was to assess TILs, TAMs and immunomodulatory targets (PD-1 and PD-L1) in the GBM microenvironment to evaluate their prognostic role in adaptive-cell-mediated immunity and to investigate their interplay with other clinicopathological and molecular features for determining GBM prognosis. In our series, we confirm the presence of T-lymphocytic infiltrate in most GBM patients (25 of 30 cases), which does not support the hypothesis that GBM is a cold tumor and suggests the presence of specific immunogenicity in GBM, as also highlighted by previous research publications [7,11,81,82]. This is an essential assumption for the future development of immunological treatments.

The percentages of CD4+ and CD8+ in our series were substantially similar to that of other case studies reported by the literature [81,83]. In our study, patients with a lower density of CD4+ lymphocytes (≤10%) had a higher survival rate than those with a higher density.

TILs have a prognostic value in a variety of cancers [20,84], generally with a survival advantage associated with the presence of cytotoxic CD8+ T cells. However, contrasting data about the association of TILs and prognosis in GBM are reported in the literature, with some studies supporting an association between an increased number of TILs and poorer prognosis [6,85], others reporting positive correlation between TILs and patients’ prognosis [19] and further studies [86] failing to find any significant association. Interestingly, Innocenti et al. reported that the presence of low CD4+ TILs combined with low CD8+ TILs is an independent predictor of longer OS [6]. A similar result has been reported in a cohort of 342 patients with malignant glioma, where the presence of T-cell infiltration in tumor tissue was associated with a poor prognosis [85]. Han et al. explored the prognostic value of CD4+ and CD8+ TILs in 90 GBM patients, concluding that a high level of CD4+ TILs combined with low CD8+ TILs was associated with lower survival.

Such different results may be due to the heterogeneity of methods used for the identification of T lymphocytes and/or for the measurement of their density and their markers of function and may certainly be explained by the inclusion in the analyses of different subgroups of gliomas, differing in grading and molecular characteristics (for example, the inclusion of lower-grade gliomas that are *IDH*-mutated) [87].

It is known that CD4+ TILs could display a double immune activity [6]: on one hand, they coordinate the immune response by stimulating the activation and the recruitment of B lymphocytes and CD8+ cells; on the other, CD4+ Tregs are key players of immune tolerance, hampering the function of effector T cells. As a consequence, despite an increase in total CD4+ TILs, the immune function of GBM patients may be impaired [6]. This might reasonably provide an explanation why, in our study, lower levels of CD4+ TILs were predictive of better OS. However, we analyzed a small cohort of patients; thus, additional research is required to confirm these results.

A limitation of the present manuscript is the absence of a multivariate assessment for the linear correlations detected due to the limited number of patients. Previous studies reported how CD8+ TILs are positively related to outcomes in other malignancies [88,89,90], and, recently, Mauldin et al. [87] confirmed this assumption in GBM. In our study, in line with the results of other series [6,91], the number of CD8+ TILs or CD4/CD8 ratio was not found to be predictive of GBM outcome.

We found MGMT-methylated tumors to have a significantly different microenvironment than unmethylated tumors. In particular, unmethylated tumors contained a substantially greater number of perivascular CD8 lymphocytes and intratumoral CD4 lymphocytes and a greater expression of CD163 in both perivascular and intratumoral sites. CD163 is a member of the scavenger receptor family and is specific for the monocyte/macrophage lineage [92,93]. We observed that CD163+ cells were present in the microenvironment of almost 100% of our GBM samples.

Macrophage polarization is a process by which macrophages assume different functional programs in response to signals from their microenvironment. This characteristic is connected with the multiple roles they play in the organism: they are powerful effector cells of the innate immune system, but they are also important in the removal of cellular debris and in tissue repair. The macrophage phenotype has been divided into two groups: CD68+ M1 classically activated macrophages and CD163+ M2 alternatively activated macrophages. This broad classification is based on in vitro studies. In addition to chemical stimuli, macrophage growth can direct its polarization state, functional roles and mode of migration. CD68+ M1 macrophages have been described as pro-inflammatory types, important in targeting host defenses against pathogens, such as the phagocytosis and secretion of pro-inflammatory cytokines and microbicidal molecules. Polarized M1 macrophages are effector cells able to induce the differentiation of lymphocytes into effector Th and to produce pro-inflammatory cytokines like IL-6 and TNF-α. Moreover, M1 macrophages produce reactive oxygen species (ROS) that mediate potent killing activity against pathogens and cancer cells [27]. CD163+ M2-polarized macrophages, instead, have been described to have a rather opposite function: resolving the acute phase of inflammation and repairing tissue damage [29,94]. M2 macrophages produce high levels of anti-inflammatory cytokines, such as IL-10 and TGF-β, and in vitro promote cancer cell invasion [95].

The widespread existence of CD163+ M2 polarized macrophages that promote tumor cell proliferation and inhibit T-cell evolution is specific to GBM [12]. Several studies have revealed that CD163+ TAM upregulation in solid tumors is associated with a short survival rate and lower responses to T-cell-based immunotherapy [96,97]; additionally, in a recent study, M2-polarized TAMs were found to be the only independent prognostic factor for gliomas among all TAMs [97]. Although our data do not demonstrate a direct correlation between the density of CD163+ macrophages and survival, their presence in almost all GBM tumor samples is a further confirmation of the importance of macrophages as a possible target of immunotherapy in high-grade gliomas [98,99]. Several research works have shown that patients with a high load of tumor-associated CD163 macrophages are less likely to survive. Targeting this subtype is much more effective than reducing the number of all macrophages. The selective depletion of CD163 in mice with melanoma caused a massive recruitment of monocytes, which matured into macrophages capable of recruiting and activating T cells, with a consequent increase in the immune response directed against the tumor [100]. This has also been tested in other cancer models, including metastatic ovarian cancer and pancreatic cancer with the same results.

In addition, we found that intratumoral CD163+ lymphocyte density increased in direct proportion to that of intratumoral CD8+ (*p* < 0.001) and intratumoral CD4+ (*p* = 0.006). This is, probably, because intratumoral macrophages recruit other tumor-promoting leukocytes [101].

PD-1/PD-L1 interactions are considered central immunological checkpoints of cancer. The rate of PD-L1-positive cases in GBM in our study was 13.3% (four cases), and nine cases were positive for PD1 (30%). These data do not appear to be consistent with the results of other studies conducted in different contexts [7], reporting that the number of PD-1-positive TILs, as well as PD-L1 expression, was significantly increased in GBM. In particular, the expression of PD1 and PD-L1 in our series appears lower than that reported in other previous studies [102]. The expression of PD-1 by T cells in malignant glioma is described to be around 50% [103]. Sobhani et al. reported a PD-L1 expression in 43% of cases of GBM [102]. Based on our analyses, PD-L1 does not seem to correlate with the OS rate of GBM patients. This result appears conflicting with the available literature. Wang et al. performed a meta-analysis of 15 studies, including a total of 108 patients, reporting a PD-L1 expression across the studies variable from 30 to 70%. They concluded that a higher expression of PD-L1 correlates with worse survival in GBM, therefore, corroborating the idea of PD-L1 as a prognostic biomarker for GBM [102,104]. Similarly, El Samman et al. performed immunohistochemical analysis on 30 GBM patients, observing PD-L1 expression in about 57% of cases and describing an association between high PD-L1 expression and poorer survival outcomes (both PFS and OS) [102,105]. Similarly, the meta-analysis by Hao et al., involving nine studies for a total of 806 GBM patients, also contributed to establishing the concept that high PD-L1 expression correlates with a worse prognosis and that PD-L1 may represent a reliable prognostic factor in GBM [102]. It cannot be excluded that our result is related to the small number of the study and to the different methods used for IHC analysis.

## 5. Conclusions

Immunotherapy has marked the breakthrough for anti-cancer treatment providing significant clinical benefit in the treatment of various solid cancers and represents a promising therapy for primary and recurrent GBM. The knowledge of the unique immune status of this tumor and the characterization of the immune cells and immune targets in GBM TME are essential to optimize future clinical trials and to successfully extend the application of immunotherapy to this rare cancer.

Our study highlighted an elevated level of TILs and TAMs in GBM *IDH*-wt microenvironment; despite the fact that, to date, no immunotherapy has been granted regulatory approval, this observation highlights the importance of developing research into innovative immunotherapeutic approaches in the future [106].

Our analysis revealed that a low number of CD4+ cells (≤10%) is inversely related to OS, whereas the number of CD8+ or CD163+ cells is not related to OS. We recognize that the sample size and the retrospective nature limit the power of survival analyses in our study. Thus, albeit of interest, these findings should be complemented by larger further studies.

We also observed that the tumor microenvironment of methylated tumors is different from that of unmethylated tumors, and this could explain their different biological behavior and also the different response to therapies, both immunotherapy and standard approaches. Furthermore, the expression of PD-L1 in GBM is confined to a subset of patients, similar to other solid cancers.

## Figures and Tables

**Table 1:** Patients clinical–pathological characteristics.

	Overall (n = 30)
**SEX**	
Female	11 (36.7%)
Male	19 (63.3%)
**SITE**	
Frontal	7 (23.3%)
Fronto-parieto-temporal	1 (3.3%)
Fronto-temporal	2 (6.7%)
Occipital	1 (3.3%)
Parietal	3 (10.0%)
Parieto-occipital	1 (3.3%)
Parieto-temporal	1 (3.3%)
Temporal	13 (43.3%)
Ventricles	1 (3.3%)
**STEROIDS DOSAGE (mg)**	
Mean (SD)	1.73 (2.07)
Median	1.38 (0, 8.00)
**MGMT**	
Unmethylated	16 (53.3%)
Methylated	14 (46.7%)
**SURGERY**	
Biopsy	1 (3.3%)
Partial resection	20 (66.7%)
Complete resection	9 (30.0%)
**BRAF**	
WT	28 (93.3%)
Mutated	2 (6.7%)
**TERT**	
WT	5 (16.7%)
Mutated	24 (80.0%)
Missing	1 (3.3%)
**P53**	
WT	18 (60.0%)
Mutated	12 (40.0%)
**PTEN**	
WT	27 (90.0%)
Mutated	2 (6.7%)
Missing	1 (3.3%)
**CD4 DENSITY**	
Negative	5 (16.7%)
≤10%	9 (30.0%)
>10%	16 (53.3%)
**CD4 DISTRIBUTION**	
Intratumoral	1 (3.3%)
Perivascular	10 (33.3%)
Peritumoral	0 (0%)
Intratumoral + perivascular	14 (46.6%)
Perivascular + peritumoral	0 (0%)
**CD8 DENSITY**	
≤10%	13 (43.3%)
>10%	5 (16.7%)
**CD8 DISTRIBUTION**	
Intratumoral	0 (0%)
Perivascular	7 (23.3%)
Peritumoral	0 (0%)
Intratumoral + perivascular	11 (36.6%)
Perivascular + peritumoral	0 (0%)
**CD163 INTRATUMORAL**	
Low	8 (26.7%)
Medium	7 (23.3%)
High	7 (23.3%)
Very High	8 (26.7%)
CD163 PERIVASCULAR	
Low	7 (23.3%)
Medium	7 (23.3%)
High	8 (26.7%)
Very High	8 (26.7%)
**PD1**	
0	21 (70.0%)
<50%	7 (23.3%)
>50%	2 (6.7%)
**PD1 DISTRIBUTION**	
Absent	21 (70.0%)
Intratumoral	3 (10.0%)
Perivascular	4 (13.3%)
Peritumoral	0 (0%)
Intratumoral + perivascular	2 (6.7%)
Perivascular + peritumoral	0 (0%)
All	0 (0%)
**PDL1**	
0	28 (93.3%)
<50%	1 (3.3%)
>50%	1 (3.3%)
**PDL1 DISTRIBUTION**	
Absent	26 (86.7%)
Intratumoral	2 (6.7%)
Perivascular	2 (6.7%)
Peritumoral	0 (0%)
Intratumoral + perivascular	0 (0%)
Perivascular + peritumoral	0 (0%)
All	0 (0%)
**TPS PDL1**	
0	26 (86.7%)
1	3 (10.0%)
2	1 (3.3%)

**Table 2:** *p*-values of linear correlations between different lymphocyte subpopulation estimated by Pearson/Spearman correlation test.

	CD4 I	CD4 P	CD8 I	CD8 P	CD163 I	CD163 P	PD-L1	Steroids
CD4 I		0.008	0.08	0.67	0.006	0.4	<0.001	0.19
CD4 P	0.008		0.62	0.002	0.31	0.86	0.008	0.79
CD8 I	0.08	0.62		0.14	<0.001	0.56	<0.001	0.11
CD8 P	0.67	0.002	0.14		0.94	0.96	0.24	0.52
CD163 I	0.006	0.31	<0.001	0.94		0.02	<0.001	0.57
CD163 P	0.4	0.86	0.56	0.96	0.02		0.34	0.17
PD_L1	<0.001	0.008	<0.001	0.24	<0.001	0.34		0.81
Steroids	0.19	0.79	0.11	0.52	0.57	0.17	0.81	

**Table 3:** Differences of lymphocyte population between MGMT methylated and unmethylated patients.

	Unmethylated (N = 21)	Methylated (N = 14)	*p*-Value
**CD4_Perivascular**			
**Mean (SD)**	301 (477)	154 (397)	0.014
**Median (Min, Max)**	75.5 (12.0, 1550)	13.0 (0, 1450)	
**Missing**	1 (4.8%)	1 (7.1%)	
**CD8_Intratumoral**			
**Mean (SD)**	20.9 (26.7)	8.85 (13.4)	0.024
**Median (Min, Max)**	14.0 (0, 125)	3.00 (0, 48.0)	
**Missing**	1 (4.8%)	1 (7.1%)	
**CD8_Perivascular**			
**Mean (SD)**	46.1 (62.5)	10.0 (12.2)	0.002
**Median (Min, Max)**	18.5 (4.00, 259)	5.00 (0, 43.0)	
**Missing**	1 (4.8%)	1 (7.1%)	

## Data Availability

The datasets used and/or analyzed during the current study are available from the corresponding author on reasonable request.
